# Deciphering and engineering the polyunsaturated fatty acid synthase pathway from eukaryotic microorganisms

**DOI:** 10.3389/fbioe.2022.1052785

**Published:** 2022-11-14

**Authors:** Pengfei Guo, Liang Dong, Fangzhong Wang, Lei Chen, Weiwen Zhang

**Affiliations:** ^1^ Laboratory of Synthetic Microbiology, School of Chemical Engineering and Technology, Tianjin University, Tianjin, China; ^2^ Key Laboratory of Systems Bioengineering and Frontier Science Center of Synthetic Biology of Ministry of Education of China, Tianjin, China; ^3^ Center for Biosafety Research and Strategy, Tianjin University, Tianjin, China

**Keywords:** polyunsaturated fatty acids, PUFA synthase, functional domain, iterative PKS, synthesis mechanisms, eukaryotes

## Abstract

Polyunsaturated fatty acids (PUFAs) are important nutrients that play important roles in human health. In eukaryotes, PUFAs can be *de novo* synthesized through two independent biosynthetic pathways: the desaturase/elongase pathway and the PUFA synthase pathway. Among them, PUFAs synthesized through the PUFA synthase pathway typically have few byproducts and require fewer reduction equivalents. In the past 2 decades, numerous studies have been carried out to identify, analyze and engineer PUFA synthases from eukaryotes. These studies showed both similarities and differences between the eukaryotic PUFA synthase pathways and those well studied in prokaryotes. For example, eukaryotic PUFA synthases contain the same domain types as those in prokaryotic PUFA synthases, but the number and arrangement of several domains are different; the basic functions of same-type domains are similar, but the properties and catalytic activities of these domains are somewhat different. To further utilize the PUFA synthase pathway in microbial cell factories and improve the productivity of PUFAs, many challenges still need to be addressed, such as incompletely elucidated PUFA synthesis mechanisms and the difficult genetic manipulation of eukaryotic hosts. In this review, we provide an updated introduction to the eukaryotic PUFA synthase pathway, summarize the functions of domains and propose the possible mechanisms of the PUFA synthesis process, and then provide future research directions to further elucidate and engineer the eukaryotic PUFA synthase pathway for the maximal benefits of humans.

## Introduction

Polyunsaturated fatty acids (PUFAs) are fatty acids with two or more double bonds in their carbon chains. In addition to helping microorganisms adapt to low-temperature environments, PUFAs are also beneficial to human health (Kawamoto et al., 2009; Yoshida et al., 2016). For example, ω3 and ω6 fatty acids, which are classified according to the position of the first double bond from the methyl end, can be used to lower the risk of cardiovascular disease (Djuricic and Calder, 2021) and have anti-inflammatory and immunomodulatory effects, since they are precursors of bioactive mediators such as prostaglandins and leukotrienes (An et al., 2018; Djuricic and Calder, 2021; Dyall et al., 2022). ω3 fatty acids also contribute to early brain and eye development in infants. However, due to the lack of a *de novo* pathway to synthesize them in human cells, PUFAs can mostly be obtained through foods (Qiu et al., 2020).

Currently, the typical sources of dietary PUFAs are marine fish, plants, oil crop seed, and oleaginous microorganisms. However, the marine fish source is unsustainable and unstable due to the issues of overfishing, climate change and environmental pollution, and the plant source depends on the season and climate change, the arable land available. Thus, microbial PUFA-producers are becoming a promising source as it is more sustainable, environmentally friendly and safe (Kothri et al., 2020; Chang et al., 2022). There are many microorganisms including bacteria, microalgae, fungi and protists that can accumulate a variety of PUFAs. Compared with bacteria, PUFA-producing eukaryotes generally accumulate higher biomass and lipids, and thus have a distinct advantage in producing PUFAs. At present, the oleaginous fungus *Mortierella alpina* is the only source for dietary arachidonic acid (ARA; 20:4 ω6) certificated by FDA and European Commission and already have been used in the food industry (Chang et al., 2021; Chang et al., 2022). The largest commercial production of microbial docosahexaenoic acid (DHA; 22:6 ω3) is produced by *Schizochytrium* species, and a significant amount of natural antioxidants (i.e. carotenoids and tocopherols) in these species can protect PUFAs from oxidation (Chi et al., 2021).

Two pathways to *de novo* synthesize PUFAs have been discovered in various organisms (Qiu et al., 2020; Chi et al., 2021). The first is the desaturase/elongase pathway, which mostly exists in eukaryotes. The oleaginous fungus *Mortierella alpina* is a representative microorganism that synthesizes PUFAs through this pathway (Chang et al., 2021; Chang et al., 2022). In the desaturase/elongase pathway, long-chain saturated fatty acids, such as palmitic acid or stearic acid, are first synthesized by fatty acid synthase (FAS) with acetyl-CoA as precursors and then catalyzed by a series of desaturases and elongases to form final PUFAs. This pathway is also called the aerobic pathway because of the requirement of molecular oxygen and has been well summarized by several recent reviews (Gong et al., 2014; Qiu et al., 2020; Remize et al., 2021; Dyall et al., 2022). The second is the PUFA synthase pathway, which is found only in various microorganisms. Different from the above pathway, the PUFA synthase pathway *de novo* synthesizes PUFAs from malonyl-CoA (Hayashi et al., 2019a) and does not require molecular oxygen; therefore, it is also called the anaerobic pathway (Qiu et al., 2020). Compared with the aerobic pathway, the anaerobic pathway has several advantages in producing PUFAs. For example, it produces fewer byproducts with undesirable chain lengths or unsaturated positions and consumes fewer reduction equivalents, NADPH (Chi et al., 2021). However, the detailed mechanisms of PUFA synthase have yet to be fully elucidated, resulting in the difficulty of producing various PUFAs at will through various metabolic engineering or synthetic biology efforts.

PUFA synthase is a multi-subunit enzyme complex and contains domains similar to FAS or PKS (polyketide synthase) (Metz et al., 2001; Qiu et al., 2020). Therefore, it is also classified as an iterative type I PKS (Chen and Du, 2016), and the corresponding pathway is called the PKS or PKS-like pathway in several studies (Blasio and Balzano, 2021; Chi et al., 2021; Jia et al., 2021). At present, the PUFAs synthesized by the PUFA synthase pathway have chain lengths of 18–24 with 2–6 *cis* carbon‒carbon double bonds, including linoleic acid (LA; 18:2 ω6) (Gemperlein et al., 2014), arachidonic acid (ARA; 20:4 ω6) (Ujihara et al., 2014), eicosapentaenoic acid (EPA; 20:5 ω3) (Metz et al., 2001), docosatetraenoic acid (DTA; 22:4 ω6) (Gemperlein et al., 2019), docosapentaenoic acid (DPA; 22:5 ω6 or ω3) (Hauvermale et al., 2006), docosahexaenoic acid (DHA; 22:6 ω3) (Hauvermale et al., 2006) and tetracosatetraenoic acid (TTA; 24:4 ω6) (Gemperlein et al., 2019), and so on. PUFA synthases have been discovered in both prokaryotes and eukaryotes, but their domain organizations are often different. As PUFA synthases have been well reviewed in several previous reviews (Hayashi et al., 2020b; Qiu et al., 2020), in this article, we focus on recent progress in PUFA synthase pathways in eukaryotes; discuss their identification, deciphering and engineering, as well as their similarities and differences with prokaryotic PUFA synthase; and provide our perspective on the future directions regarding the use of eukaryotic PUFA synthase pathways.

## Identification of PUFA synthases in eukaryotes

Metz et al. (Metz et al., 2001) first described the eukaryotic PUFA synthase pathway and found that the synthesis of DHA and DPA does not depend on membrane-bound elongases and desaturases in *Schizochytrium* sp. ATCC 20888 (*Schizochytrium* sp. S31), and three ORFs discovered from the *Schizochytrium* cDNA library exhibit homology to the PUFA synthase of *Shewanella*. Hauvermale et al. (Hauvermale et al., 2006) later expressed these genes in *Escherichia coli*, resulting in the production of a similar ratio of DPA and DHA in *Schizochytrium* sp. ATCC 20888. *Schizochytrium* strains with the *orfA*, *orfB* or *orfC* genes deleted could only grow in the presence of supplemented PUFAs, which demonstrates that each gene is important in PUFA synthesis (Lippmeier et al., 2009). At present, homologous genes of PUFA synthase have been identified in many eukaryotes, especially in thraustochytrids, but only a few have been functionally verified by heterologous expression (Table 1).

**TABLE 1:** Identification of PUFA synthases in eukaryotes.

Species	Products	Identification methods	References
*Schizochytrium* sp. ATCC20888	DHA, ω6 DPA	Heterologous expression in *E. coli*	Hauvermale et al. (2006)
*Thraustochytrium* sp. 26185	DHA, ω6 DPA	Heterologous expression in *E. coli*	Meesapyodsuk and Qiu, (2016)
*Schizochytrium* sp. ATCC PTA-9695	DHA, EPA	Heterologous expression in *Arabidopsis*	(Walsh et al., 2016; Sidorenko et al., 2017)
*Aurantiochytrium* sp. OH4	DHA, ω6 DPA	Heterologous expression in *E. coli*	Hayashi et al. (2019a)
*Aurantiochytrium* sp. SD116	DHA, ω6 DPA	Heterologous expression in *E. coli*	Wang et al. (2020a)
*Schizochytrium* sp. FJU-512	DHA, ω6 DPA	cDNA analysis	Huang et al. (2008)
*Schizochytrium mangrovei* PQ6	DHA, ω6 DPA	Transcriptomic analysis	Hoang et al. (2016)
*Schizochytrium* sp. HX-308	DHA, DPA	Transcriptomic analysis	Ren et al. (2017)
*Schizochytrium* sp. TIO01	DHA, ω6 DPA	Genomic analysis	Hu et al. (2020)
*Aurantiochytrium* sp. L-BL10	—	mRNA analysis	GenBank Accession: AIJ29322.1, AIJ29323.1, AIJ29324.1
*Aurantiochytrium* sp. T66	DHA, DPA	Genomic analysis	Heggeset et al. (2019)
*Aurantiochytrium* sp. PKU#SW7	DHA	Transcriptomic analysis	Liang et al. (2018)
*Hondaea fermentalgiana* CCAP 4062/3	DHA, ω6 DPA	Genomic analysis	Dellero et al. (2018)
*Schizochytrium limacinum* B4D1	DHA, ω6 DPA	Genomic analysis	Zhang et al. (2017)
*Schizochytrium limacinum* SR21	DHA, DPA	Genomic analysis, heterologous expression of *orfC* in *S. cerevisiae*	(Liang et al., 2020; Shi et al., 2021)
*Alexandrium minutum*	DHA, EPA	^13^C-Labelling analysis	Remize et al. (2020)
*Thraustochytriidae* sp. PKU#SW8	DHA, ω6 DPA	Heterologous expression of *orfB* in *E. coli*	Chen et al. (2020)
*Thraustochytrium aureum* ATCC34304	DHA, ω6 DPA	Gene disruption	Matsuda et al. (2012)
*Aurantiochytrium limacinum* OUC168	DHA, DPA	Genomic analysis	Liu et al. (2018)
			GenBank Accession: MH636606.1, JX096393.1,
			JX096392.1
*Aurantiochytrium* SW1	DHA, ω6 DPA	Separation and identification of proteins	Shuib et al. (2022)

**FIGURE 1:**
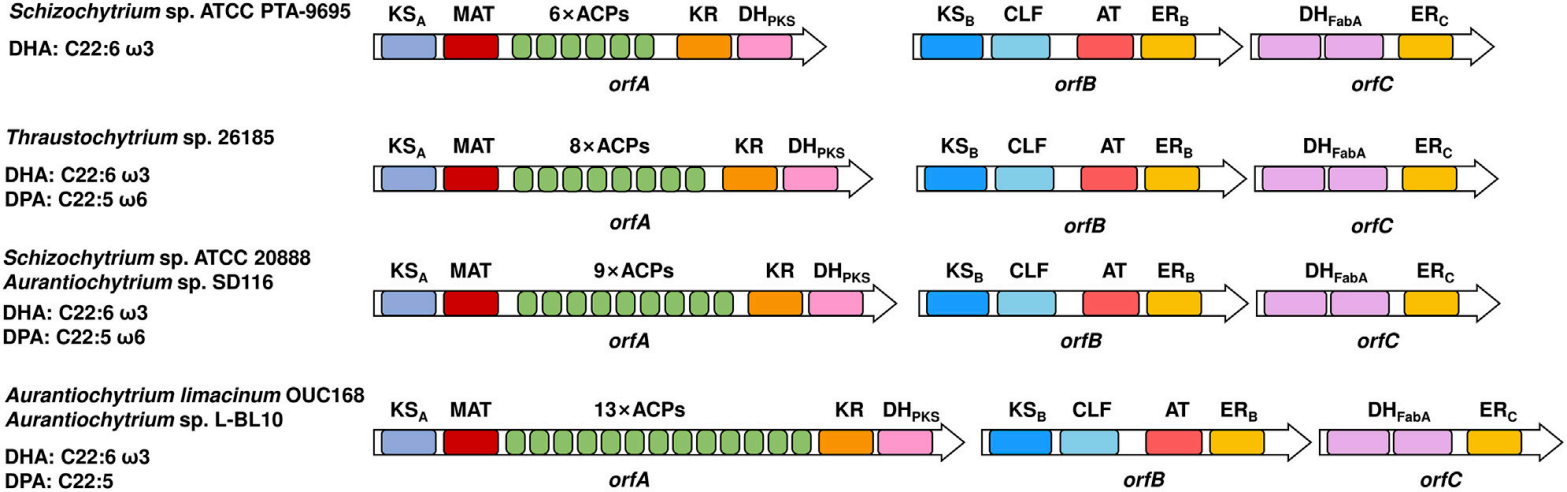
Domain organizations of eukaryotic PUFA synthases. On the left are the native sources and main products of PUFA synthases. Different domains are indicated by colors. ACP, acyl carrier protein domain; MAT, malonyl-CoA: ACP transacylase domain; KS, ketoacyl-ACP synthase domain; CLF, chain length factor domain; KR, ketoacyl-ACP reductase domain; DH_PKS_, PKS-like dehydratase domain; DH_FabA_, FabA-like dehydratase domain; ER, enoyl-ACP reductase domain; AT, acyltransferase domain. The subscripts A, B and C indicate that the corresponding domain is located in subunits A, B and C, respectively.

All eukaryotic PUFA synthases that have been identified thus far are composed of three subunits, subunit A, subunit B and subunit C, which are encoded by *orfA*, *orfB* and *orfC*, respectively (Figure 1). From the N- to C-terminus, the domain arrangement of subunit A is the ketoacyl-ACP synthase (KS_A_) domain, malonyl-CoA: ACP transacylase (MAT) domain, tandem acyl carrier protein (ACP) domains and ketoacyl-ACP reductase (KR) domain, and PKS-like dehydratase (DH_PKS_) domain. It is KS_B_, chain length factor (CLF) domain, acyltransferase (AT) domain and enoyl-ACP reductase (ER) domain in subunit B, and two tandem FabA-like dehydratase (DH_FabA_) domains and ER_C_ domain in subunit C. The domain organization of each subunit shows little difference among different eukaryotic species.

## Function of domains in eukaryotic PUFA synthase

### Tandem acyl carrier protein domains

ACP domains exist in 6–13 tandem forms in eukaryotic PUFA synthase, which function as loading acyl groups in the biosynthesis of PUFAs. The serine residue at the active sites of ACP is modified by phosphopantetheinyl transferase (PPTase) to transform *apo*-ACP into *holo*-ACP with a 4′-phosphopantetheine (Ppant) arm (Beld et al., 2014; Rullan-Lind et al., 2019). Then, the fatty acyl chain is covalently connected with *holo*-ACP through thioester bond and catalyzed by different domains to complete the PUFA synthesis process. Three genes encoding PUFA synthase from *Schizochytrium* sp. ATCC 20888 and a *hetI* encoding PPTase from *Nostoc* sp. PCC 7120 were heterologously co-expressed in *E. coli* (Hayashi et al., 2016). It was found that the number of ACP units in subunit A was positively correlated with the productivity of PUFAs, as the deletion of ACP domains led to a decrease in PUFA productivity and the insertion of ACP domains or inactivating ACP domains led to increased productivity (Hayashi et al., 2016). Each ACP of *Shewanella japonica* PUFA synthase can be activated by PPTase (Jiang et al., 2008), but this characteristic has not been confirmed in eukaryotic PUFA synthases. PPTases are not present in eukaryotic PUFA synthases but are found elsewhere in the genomes of *Thraustochytrium* sp. 26185, *Schizochytrium* sp. ATCC MYA-1381 (*S. limacinum* SR21) (Meesapyodsuk and Qiu, 2016) and *Aurantiochytrium* sp. SD116 (Wang et al., 2020a). Previous studies also showed that the overexpression of endogenous PPTase significantly increased the proportion of PUFAs, especially DHA, in *Aurantiochytrium* (Wang et al., 2020a).

### Ketoacyl-ACP synthase domain

There are two ketoacyl-ACP synthase (KS) domains in eukaryotic PUFA synthase. One is at the N-terminus of subunit A, named KS_A_, and the other is KS_B_ at the N-terminus of subunit B, which is adjacent to a CLF domain. The KS domain catalyzes the Claisen condensation reaction of the extender unit and acyl-ACP, leading to a two-carbon extension in the carbon chain (Hayashi et al., 2016; Yoshida et al., 2016; Xie et al., 2017). It has been found that both the KS_A_ and KS_B_ of *Thraustochytrium* sp. strain ATCC 26185 could complement the defective phenotypes of both ketoacyl-ACP synthase I (FabB) and ketoacyl-ACP synthase II (FabF) mutants in *E. coli*, suggesting their ketoacyl-ACP synthase activities (Xie et al., 2017). In prokaryotic PUFA synthase, the KS domain adjacent to the CLF domain catalyzes the last elongation step for DHA biosynthesis, and two KS domains have selectivity for the recognition of chain length and the degree of unsaturation of acyl-ACP intermediates (Hayashi et al., 2019a; Santín et al., 2020). It is likely that eukaryotic KS domains have similar characteristics. Hayashi et al. found that mutation of three amino acids located in the KS_B_ of *Aurantiochytrium* sp. OH4 could change the major product from DHA (EPA/DHA = 0) to EPA (EPA/DHA=1.07) in *E. coli* (Hayashi et al., 2019a). However, the substrate selectivity of the two KS domains in eukaryotic PUFA synthase still needs to be further investigated.

In addition, the CLF domain in PUFA synthase shares high sequence similarity with the KS domain, but it lacks conserved active residues (Hayashi et al., 2019a) and cannot form a covalent bond with the acyl group. In prokaryotic PUFA synthase, the CLF domain and its adjacent KS domain are usually studied as a whole (Hayashi et al., 2019a; Santín et al., 2020). It has been demonstrated that the CLF domain is the primary determinant of polyketide chain length in polyketide biosynthesis (Tang et al., 2003). The disruption of the CLF domain in *Schizochytrium* sp. ATCC MYA-1381 (*S. limacinum* SR21) affected growth, decreased the content of PUFAs and increased the content of SFAs, and the proportion of C22 PUFA decreased by 57.51% (Li et al., 2018).

### Acyltransferase-like domain

Eukaryotic PUFA synthase contains two AT-like domains, the malonyl-CoA: ACP transacylase (MAT) domain in subunit A and the acyltransferase (AT) domain in subunit B. The MAT domain catalyzes the binding of the malonyl group and ACP, and the AT domain has thioesterase activity and catalyzes the release of free fatty acids from long-chain acyl-ACPs. Complementation tests showed that the MAT domain of *Thraustochytrium* sp. 26185 was able to restore the growth phenotype of a temperature-sensitive *E. coli* mutant defective in malonyl-CoA: ACP transacylase (FabD) activity, but the AT domain could not (Almendariz-Palacios et al., 2021). *In vitro* experiments show that the AT domain has thioesterase activity against fatty acyl-ACPs and fatty acyl-CoAs (Hayashi et al., 2020a; Almendariz-Palacios et al., 2021). The AT domain of *Schizochytrium* tends to catalyze DHA-ACP, EPA-ACP and long saturated acyl-ACPs among fatty acyl-ACP substrates; the AT domain of *Thraustochytrium* shows higher activity toward DHA-CoA among fatty acyl-CoA substrates, and mutation of two putative active site residues S96 or H220 at the AT domain resulted in reduced catalytic activity toward DHA-CoA (Hayashi et al., 2020a; Almendariz-Palacios et al., 2021). These results suggest the AT domain might be involved in the release of freshly synthesized DHA as free fatty acid from the PUFA synthase. In addition, the overexpression of the AT domain increased the free fatty acid content in the *E. coli* mutant with the defective β-oxidation-related gene *fadD* (Almendariz-Palacios et al., 2021). The deletion of the AT domain led to a slow cell growth rate and reduced content of DHA, but the complementary strain with the AT domain from *Shewanella* exhibited the recovery of growth and increased content of EPA and DHA in *Schizochytrium* sp. HX-308 (Ren et al., 2018).

### Ketoacyl-ACP reductase domain

There is a KR domain located in subunit A. The KR domain imbedded in prokaryotic PUFA synthase is able to reduce 3-oxoacyl-ACP to (*3R*)-hydroxyacyl-ACP in the presence of NADPH (Hayashi et al., 2019b), and the function of the KR domain in eukaryotic PUFA synthase is probably the same, although this requires further confirmation. In addition, overexpression of the KR domain led to an increase in biomass, total fatty acid content and DHA content in *A. limacinum* OUC168 (Liu et al., 2018).

### Dehydratase domain

Eukaryotic PUFA synthase contains three DH domains. The sequence of the DH domain at the C-terminus of subunit A is similar to that in PKS synthase, which is named DH_PKS_. The other two domains exhibit higher similarity with β-hydroxyacyl-ACP dehydratase (FabA) from *E. coli* and are named DH_FabA_. All three domains can complement the defective phenotype of the *E. coli fabA* temperature-sensitive mutant, suggesting that they can function as β-hydroxyacyl-ACP dehydratases (Xie et al., 2018). In prokaryotic PUFA synthase, both DH_PKS_ and DH_FabA_ catalyze the dehydration of β-hydroxyacyl-ACP but two kinds of DH domains have selectivity toward substrates with different chain lengths and DH_FabA_ domains also have the ability to catalyze 2,2- and 2,3-isomerization reactions (Hayashi et al., 2019b).

The DH domains in eukaryotic PUFA synthase may have the same characteristics, which still needs to be confirmed. Man et al. showed that heterologous expression of two DH_FabA_ domains can increase the proportion of unsaturated fatty acids, while heterologous expression of DH_PKS_ helps increase saturated fatty acids (Man et al., 2021) in *E. coli*. Overexpression of DH_PKS_ in *A. limacinum* OUC168 showed increased contents of both C20:4 and total fatty acids (Liu et al., 2018). Heterologous expression of DH_PKS_ and DH_FabA_ domains in *E. coli* increased the biomass and weakened the inhibitory effect of mid-chain fatty acids on cell growth (Man et al., 2021). In addition, Xie et al. (Xie et al., 2020) pointed out that there are some differences in terms of the functions of these two DH domains, DH_FabA_-1 and DH_FabA_-2. The domain swapping analysis showed that only the DH_FabA_-1 domain can functionally replace the DH domain of a type I fatty acid synthase in yeast. The heterologous expression of PUFA synthase in *E. coli* showed that the mutation or deletion of DH_FabA_-1 or substitution of DH_FabA_-1 with DH_FabA_-2 led to the loss of PUFA production capacity, and the mutation or deletion of DH_FabA_-2 led to a small amount of DPA production and no DHA production (Xie et al., 2020). The overexpression of the DH_FabA_-1 domain in *S. limacinum* SR21 increased the content of PUFAs and total lipids (Shi et al., 2021). Disruption of the DH_FabA_-2 domain of *Schizochytrium* sp. ATCC MYA-1381 (*S. limacinum* SR21) affected cell growth, and the proportion of PUFAs was decreased, while the proportion of saturated fatty acids (SFAs) was increased (Li et al., 2018). In a heterologous expression of protist PUFA synthase in canola seeds, Walsh et al. mentioned that the ratio of DHA to DPA was increased by replacing DH_FabA_-2 of *Schizochytrium* with DH_FabA_-2 of *Thraustochytrium* sp. 23B (Walsh et al., 2016).

### Enoyl-ACP reductase domain

Enoyl-ACP reductases catalyze the reduction of 2-*trans* enoyl-ACPs to form α, β-saturated acyl-ACPs in fatty acid biosynthesis (Massengo-Tiassé and Cronan, 2009). There are two ER domains in eukaryotic PUFA synthase. The ER_B_ or ER_C_ domain is located at the C-terminus of subunit B or C, respectively. Knockout of ER_B_ significantly decreased the content of PUFAs in *S. limacinum* SR21. Knockout of ER_C_ reduced the content of SFAs, while overexpression of ER_C_ led to a decrease in PUFA content and an increase in SFA content in *S. limacinum* SR21 (Ling et al., 2020; Shi et al., 2021). In addition, several lines of evidence have demonstrated that the addition of triclosan can inhibit the expression of ER_C_ and lead to an increase in PUFA production (Ling et al., 2020). These results suggest that ER_B_ plays an important role in the synthesis of PUFAs, and ER_C_ is more likely to be related to the synthesis of SFAs in *S. limacinum* SR21. When ER_B_ of *S. limacinum* SR21 was replaced by the ER domain of *Shewanella* SCRC2738, the content of EPA was increased by 85.7%, and the transcriptional level of several genes of PUFA synthase was also upregulated. However, there was little effect on the synthesis of PUFAs when ER_C_ was replaced by ER of *Shewanella* SCRC2738 (Yang et al., 2021).

## Mechanism analysis of the eukaryotic PUFA synthase pathway

**FIGURE 2:**
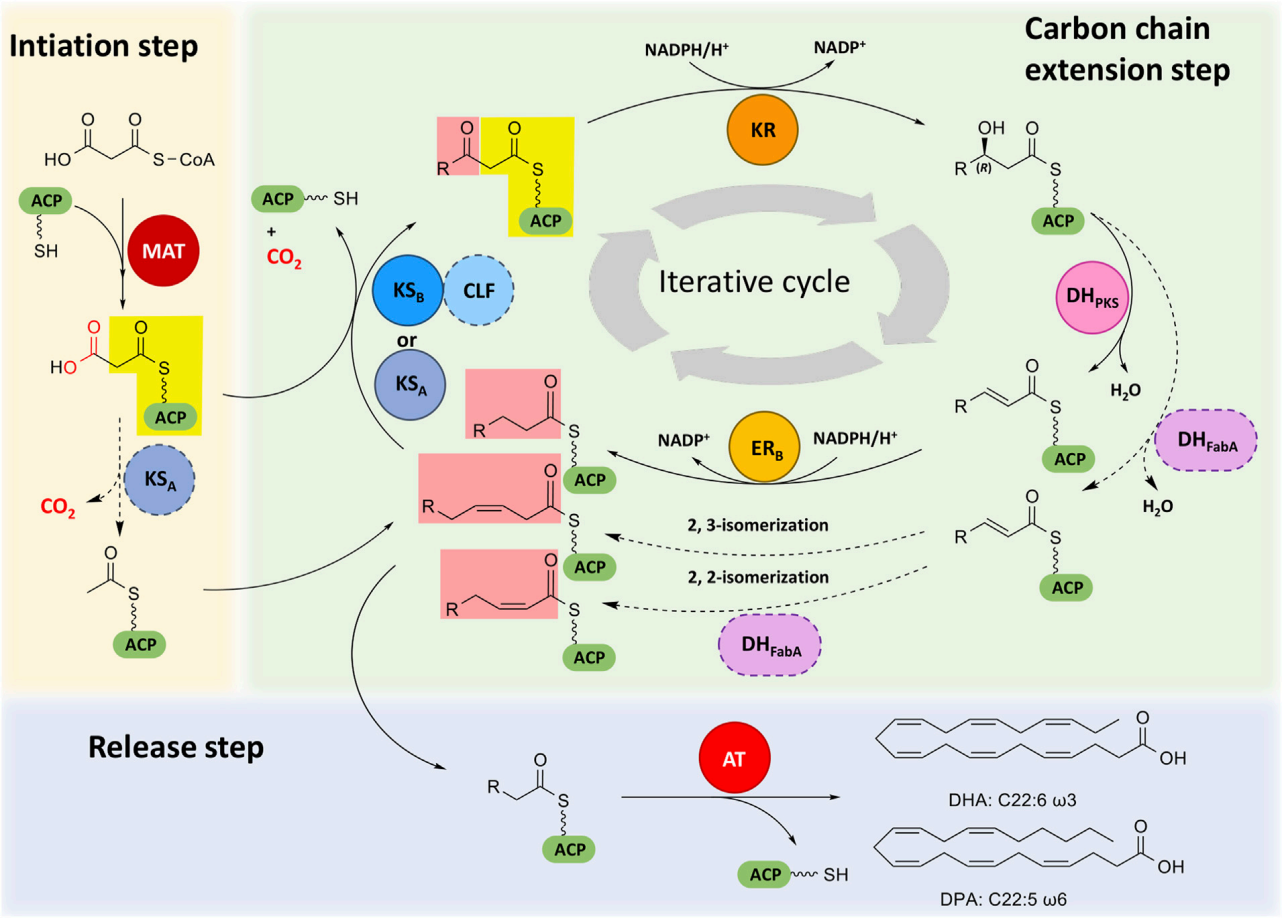
Proposed PUFA biosynthetic machinery in eukaryotic PUFA synthases. Different domains are indicated by colors. ACP, acyl carrier protein domain; MAT, malonyl-CoA: ACP transacylase domain; KS, ketoacyl-ACP synthase domain; CLF, chain length factor domain; KR, ketoacyl-ACP reductase domain; DH_PKS_, PKS-like dehydratase domain; DH_FabA_, FabA-like dehydratase domain; ER, enoyl-ACP reductase domain; AT, acyltransferase domain. Dotted arrows indicate possible reactions, and domains with dashed outlines indicate their possible involvement in related reactions. The atoms lost in the decarboxylation of malonyl-ACP are shown in red. Different background colors are given to different parts of β-ketoacyl-ACP formed by condensation.

Elucidating the mechanisms of PUFA synthesis mediated by PUFA synthase has always been a hot topic. At present, the mechanistic analysis of prokaryotic PUFA synthases goes further than that of eukaryotic PUFA synthases (Napier, 2002; Hayashi et al., 2020a; Hayashi et al., 2020b). Eukaryotic PUFA synthases contain the same domain types as prokaryotic PUFA synthases, although the number and arrangement of domains in each subunit are different. Therefore, the mechanisms for synthesizing PUFAs by these two kinds of PUFA synthases may be similar to some extent. Here, we divided the process of eukaryotic PUFA synthesis into three steps (Figure 2), and the possible synthesis mechanism related to each step is described.

### Initiation step

The initiation of PUFA synthesis involves the synthesis of acetyl-ACP and malonyl-ACP. MAT, KS, tandem ACP domains and PPTase participate in this process. The tandem ACPs are activated by PPTase from *apo*-ACPs to *holo*-ACPs. In the prokaryotic PUFA synthase pathway, the MAT domain specifically recognizes and catalyzes the loading of malonyl-CoA onto *holo*-ACP to form malonyl-ACP (Santin and Moncalian, 2018), and then, the KS_A_ domain catalyzes the decarboxylation of malonyl-ACP to form acetyl-ACP (Hayashi et al., 2019a; Santín et al., 2020). Similarly, the MAT domain of eukaryotic PUFA synthase has the function of malonyl-CoA: ACP transacylase (Almendariz-Palacios et al., 2021), which transfers the malonyl moiety from malonyl-CoA onto ACP, but its substrate specificity has not been evaluated. *In vitro* experiments also suggested that the starter unit acetyl-ACP could be derived from malonyl-ACP in the PUFA synthesis process of *Schizochytrium* (Metz et al., 2009). Malonyl-ACP is used as the carbon chain extender unit during the subsequent carbon chain extension process.

### Carbon chain extension step

The carbon chain extension step of PUFA synthesis involves the elongation of fatty acyl-ACPs and the formation of *cis* double bonds at the appropriate position. KS, CLF, tandem ACPs, KR, DH_PKS_, DH_FabA_ and ER domains are involved. It is widely accepted that the carbon chain extension of the PUFA synthase pathway is an iterative process, which is the same as the type I fatty acid synthesis pathway or iterative PKS pathway (Hayashi et al., 2020b; Qiu et al., 2020; Remize et al., 2021). Each iterative process elongates two carbon chains and forms saturated or *cis* double bonds at the appropriate positions, but the details have not been well elucidated until now.

In the prokaryotic PUFA synthase pathway, the iterative process is speculated to be as follows. First, fatty acyl-ACP is condensed with malonyl-ACP to form β-ketoacyl-ACP through catalysis of the KS domain. Two KS domains have selectivity for the chain length and the degree of unsaturation of acyl-ACPs. The KS_A_ domain is responsible for the elongation of short chain (C2, C4, C6, C10) and long chain (C16, C18) substrates, and the KS-CLF didomain is responsible for the elongation of medium chain (C10) and very long chain C20 substrates (Hayashi et al., 2019a; Santín et al., 2020). Second, the KR domain is responsible for reducing the extended β-ketoacyl-ACP to (*3R*)-hydroxyacyl-ACP (Hayashi et al., 2019b). Then, (*3R*)-hydroxyacyl-ACP is dehydrated by the DH domain to form an α, β-*trans* double bond, but the difference between DH_PKS_ and DH_FabA_ leads to distinct subsequent reactions. When the *trans* double bond is formed under the dehydration of DH_PKS_, the next reaction will be the reduction by the ER domain and form an α, β-saturated bond. When the *trans* double bond is formed under the dehydration of DH_FabA_, it will be further catalyzed to form a *cis* double bond by 2,2- or 2,3-isomerization (Hayashi et al., 2019b). After several iterations, the extension of fatty acyl-ACP was finished, forming PUFA-ACP.

In eukaryotes, the iterative process is likely the same as that in prokaryotes, which comprises condensation of acyl-ACPs and extender units, reduction of β-ketoacyl-ACPs, dehydration of β-hydroxyacyl-ACPs and selective reduction of enyl-ACPs. However, as the sequence, number and location of KS, DH and ER are obviously different from those in prokaryotes, it is speculated that the details of carbon chain elongation and double bond formation could also be different from those in prokaryotes, although details still need further investigation.

### Release step

The release process of PUFAs involves the offloading of the fatty acyl group from ACP, which is finished by the AT domain in subunit B. Catalyzed by the AT domain, a specific long chain fatty acyl group linked with the thioester bond is hydrolyzed from the Ppant arm of *holo*-ACP, and the final PUFA is released as a free fatty acid (Metz et al., 2009; Hayashi et al., 2020a).

## Similarities and differences between prokaryotic and eukaryotic PUFA synthase pathways

### Similarities

First, both are independent of the FAS pathway, and PUFAs can be synthesized *de novo* from malonyl-CoA using NADPH as a cofactor (Metz et al., 2009) and released in the form of free fatty acids. Second, the PUFA synthases of eukaryotes and prokaryotes contain the same types of domains, and the basic functions of these domains are also similar (Qiu et al., 2020). Last, the main methodologies used in studies on the PUFA synthase pathway of prokaryotes and eukaryotes are basically the same, including *in vitro* enzyme reactions and *in vivo* experiments such as heterologous expression, deletion, addition and replacement of subunits or domains.

### Differences

**FIGURE 3:**
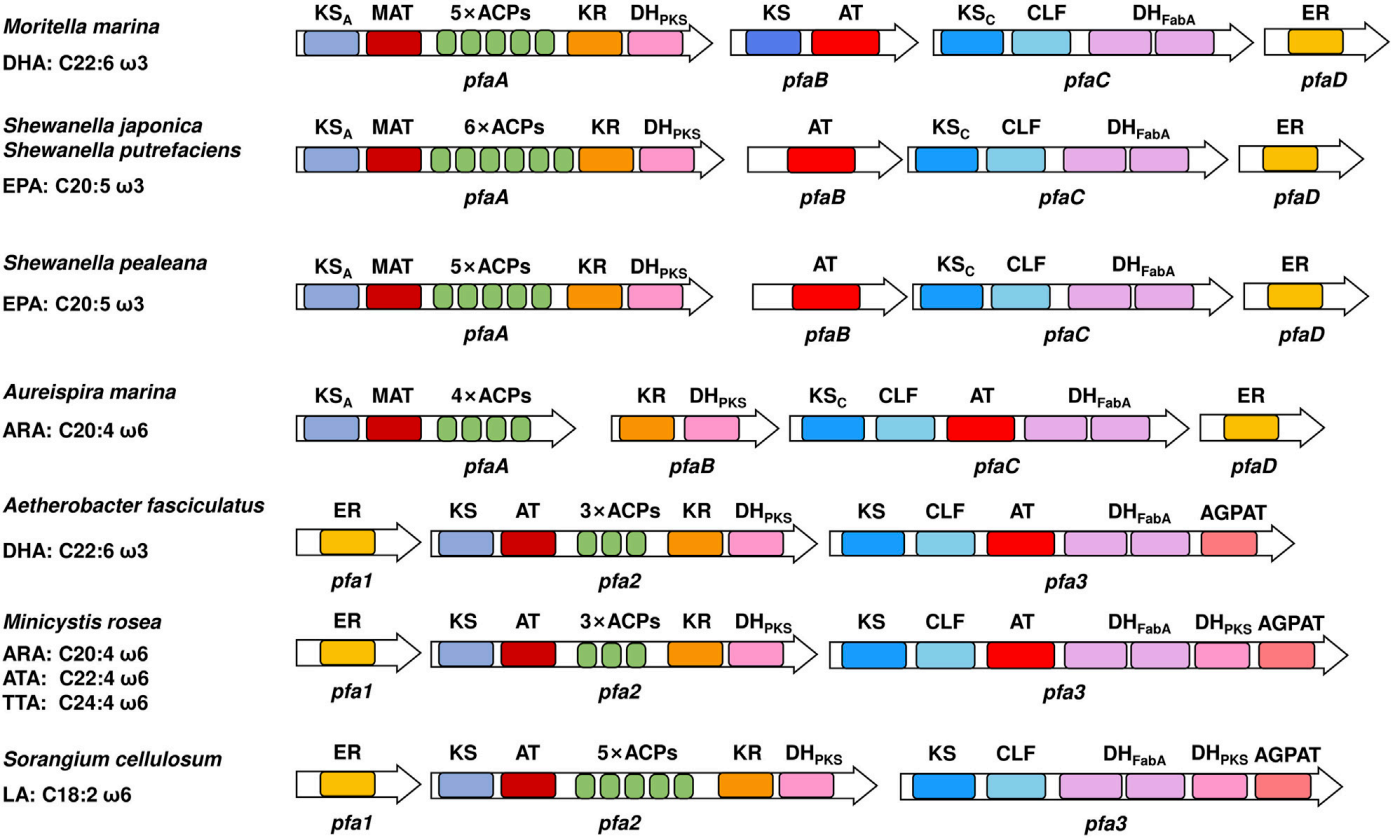
Domain organizations of several prokaryotic PUFA synthases. On the left are the native sources and main products of PUFA synthases. Different domains are indicated by colors. ACP, acyl carrier protein domain; MAT, malonyl-CoA: ACP transacylase domain; KS, ketoacyl-ACP synthase domain; CLF, chain length factor domain; KR, ketoacyl-ACP reductase domain; DH_PKS_, PKS-like dehydratase domain; DH_FabA_, FabA-like dehydratase domain; ER, enoyl-ACP reductase domain; AT, acyltransferase domain; AGPAT, 1-acylglycerol-3-phosphate *O*-acyltransferase domain. The subscripts A and C indicate that the corresponding domain is located in subunits A and C, respectively.

First, the major product synthesized by the PUFA synthase pathway in eukaryotes is DHA, while it is EPA, DHA, ARA or LA in prokaryotes (Gemperlein et al., 2014; Morabito et al., 2019). Second, the number of ER domains and the domain organizations of PUFA synthases of eukaryotes and prokaryotes are different (Qiu et al., 2020). Third, there are some differences in the properties and catalytic activities of the domains. For example, two KS domains in prokaryotic PUFA synthase could not be expressed in the form of a single enzyme and were studied in the form of KS-AT and KS-CLF didomains (Hayashi et al., 2019a; Santín et al., 2020). However, in *Thraustochytrium* sp., both KS domains were expressed as stand-alone enzymes in *E. coli* (Xie et al., 2017). The AT domain of *Schizochytrium* sp. exhibited strong substrate selectivity, while the AT domain in *P. profundum* showed promiscuous substrate specificity against short to long acyl chains (Hayashi et al., 2020a). Moreover, since a special 1-acylglycerol-3-phosphate *O*-acyltransferase (AGPAT) domain exists in the PUFA synthase of myxobacteria (Gemperlein et al., 2014), it is speculated that there might be other PUFA release mechanisms by directly forming glycerol phospholipids in prokaryotes, while it has never been found in eukaryotes. Last, studies on these two pathways typically have different focuses. The diversity of major products synthesized by prokaryotic PUFA synthases suggests that there are differences in catalytic activity of some domains (Figure 3), which makes it relatively easy to study the catalytic mechanism. Thus, studies of prokaryotic PUFA synthases mainly focus on mechanism research, such as elucidation of the structure and function of domains. In contrast, eukaryotic microorganisms such as *Schizochytrium* have already been used as industrial strains for the production of DHA-rich oils (Chi et al., 2021), therefore most studies of eukaryotic PUFA synthase pathways focus on application research, such as changes in the profiles and contents of PUFA products (Table 2). Similarities and differences between prokaryotic and eukaryotic PUFA synthase pathways.

**TABLE 2:** Similarities and differences between prokaryotic and eukaryotic PUFA synthase pathways.

Similarities	Differenties		
		Prokaryotic PUFA synthase pathway	Eukaryotic PUFA synthase pathway
Independent of the FAS pathway; *de novo* synthesize PUFAs from malonyl-CoA; use NADPH as a cofactor; PUFAs are released as free fatty acids	The major product	EPA	DHA
		DHA	
		ARA	
		LA	
Contain the same types of domains; the basic functions of domains are similar	The number and organization of the domains	An ER domain is located in a separate subunit; some subunits contain only one domain	Contains two ER domains; each subunit contains multiple domains
—	The properties and catalytic activities of the domains	KS domains cannot be expressed as stand-alone enzymes	KS domains can be expressed as stand-alone enzymes
The study methods are basically the same	The study focus	Mechanism research	Application research

## Challenges and future perspectives

Current progress has given us a preliminary understanding of the eukaryotic PUFA synthase pathway, but there are still many challenges in elucidating the entire PUFA synthesis process and expanding the future applications of the pathway: 1) Eukaryotic PUFA synthases have been mainly identified in thraustochytrids, and the major products are all DHA, which limits their applications and hinders the functional comparative research on eukaryotic PUFA synthases; 2) The structure and function of each domain of eukaryotic PUFA synthases have not been fully revealed, which makes it difficult to elucidate the biosynthetic logic of PUFAs and brings difficulties to the subsequent optimization and modification of eukaryotic PUFA synthases; 3) The natural eukaryotic PUFA synthases discovered thus far are difficult to achieve mass production of specific PUFAs, and the modification of PUFA synthases is an effective way to achieve this goal, but compared with other iterative PKS synthases, there are few studies on the engineering of PUFA synthases; 4) The genetic transformation of eukaryotic microbes, such as protists, with the PUFA synthase pathway is still difficult, and the lack of efficient transformation methods and genome manipulation tools increases the complexity of researching the PUFA synthase mechanism *in vivo*, thus making it difficult to perform metabolic engineering. In response to these challenges, future research needs to be carried out from the following perspectives.

### Identification and validation of more PUFA synthases

Different from those in eukaryotes, prokaryotic PUFA synthases have been found in many marine bacteria and even myxobacteria, and the main PUFA products produced by these PUFA synthases are more diverse. Table 3 summarizes the prokaryotes with PUFA synthase that have been heterogeneously expressed to date.

**TABLE 3:** Prokaryotic PUFA synthases identified by heterologous expression.

Main PUFA product	Organisms	Chassis	References
EPA	*Shewanella japonica*	*E. coli*	Jiang et al. (2008)
	*Shewanella oneidensis* MR-1	*E. coli*	(Lee et al. (2006); Hayashi et al. (2016))
	*Photobacterium profundum* SS9	*E. coli*	Hayashi et al. (2019a)
	*Shewanella putrefaciens* SCRC-2738	*E. coli*	Metz et al. (2001)
	*Shewanella baltica* MAC1	*E. coli* and *Lactococcus lactis*	(Amiri-Jami and Griffiths, (2010); Amiri-Jami et al. (2014))
	*Shewanella* sp. BR-2	*E. coli*	Lee et al. (2009)
	*Shewanella pealeana* ATCC 700345	*E. coli*	Allemann et al. (2019)
DHA, ω3 DPA	*Moritella marina* MP-1	*E. coli*	Orikasa et al. (2006)
DHA	*Colwellia psychrerythraea* 34H	*E. coli*	Peng et al. (2016)
	*Aetherobacter fasciculatus*	*Myxococcus xanthus* DK1622; *E. coli*; *Pseudomonas putida* KT2440	(Gemperlein et al. (2014); Gemperlein et al. (2016))
DHA, EPA	*Aetherobacter* sp.	*Myxococcus xanthus* DK1622	Gemperlein et al. (2014)
ARA, DTA (22:4 ω6), TTA (24:4 ω6)	*Minicystis rosea*	*Yarrowia lipolytica*	Gemperlein et al. (2019)
ARA	*Aureispira marina*	*E. coli*	(Ujihara et al. (2014); Hayashi et al. (2016))
LA	*Sorangium cellulosum* So ce56	*Myxococcus xanthus* DK1622	Gemperlein et al. (2014)

In addition to the diverse PUFA profiles, the domain organizations between some prokaryotic PUFA synthases are also quite different (Figure 3), which provides good candidates for the studies of prokaryotic PUFA synthases. At present, more PUFA-producing eukaryotic microorganisms are being discovered (Colonia et al., 2021; Kalidasan et al., 2021; Wang et al., 2021; Bai et al., 2022), and species with differentiated PUFA profiles are likely to be discovered in the future. Analyzing the genomes of these species and expressing the possible genes in *E. coli* will be an efficient way to identify novel PUFA synthases. The discovery and identification of distinct PUFA synthases in more eukaryotic species is of great value for both mechanistic research and engineering of eukaryotic PUFA synthases.

### Functional identification of domains

As mentioned above, functional research on each domain can help elucidate the biosynthesis process of PUFAs; for example, the study of the MAT domain can reveal the initiation of PUFA synthesis, the study of the KS, KR, DH, and ER domains can reveal the iterative process of PUFA synthesis, and the study of the AT domain helps us understand the release of PUFAs. Since AT-like, KS, ACP, DH, and ER domains have multiple copies in eukaryotic PUFA synthases, it is necessary to determine whether there are functional differences between homologous domains. Referring to the existing research of PUFA synthase and other multidomain enzymes, the functional research of eukaryotic PUFA synthase domains can be expanded through *in vivo*, *in vitro* and structural analysis.

#### *In vivo* assay

There have been some studies utilizing overexpression or inactivation of domains to study their possible function and their contribution to PUFA synthesis. For example, the addition and inactivation of ACP domains have been performed in *E. coli* expressing eukaryotic PUFA synthase (Hayashi et al., 2016); DH_PKS_, DH_FabA_-1, ER_C_ and KR domains have been overexpressed in their native hosts (Liu et al., 2018; Shi et al., 2021), and DH_FabA_-2, CLF, AT and two ER domains have been knocked out (Li et al., 2018; Ren et al., 2018; Ling et al., 2020). Therefore, the functions of the remaining undetermined domains could also be analyzed *in vivo* using both overexpression and knockout methods in the future.

In addition, heterologous expression of unknown genes and their functional complementation assays of well-studied enzymes in organisms with clear genetic backgrounds can help determine their functions. This method is also applicable to the study of eukaryotic PUFA synthase. For example, by complementing the function of ketoacyl-ACP synthase in *E. coli*, it has been demonstrated that both KS domains possess the activities of ketoacyl-ACP synthase (Xie et al., 2017); the functional complementation of dehydratase in *E. coli* indicates that all three DH domains possess dehydratase activities (Xie et al., 2018), but only DH_FabA_-1 of two DH_FabA_ domains can functionally replace the DH domain of a type I fatty acid synthase in yeast (Xie et al., 2020); the functional replacement of MAT in *E. coli* indicates that only the MAT domain located in subunit A has the function of MAT (Almendariz-Palacios et al., 2021). Similarly, the KR domain and two ER domains may also be probed for functions through functional complementarity of corresponding discrete enzymes.

#### *In vitro* assay

*In vitro* assay is an intuitive and convincing approach to analyze the function of enzymes. In studies of prokaryotic PUFA synthases, the catalytic activities of the KS domains (Hayashi et al., 2019a; Santín et al., 2020), DH domains (Hayashi et al., 2019b), KR domain (Hayashi et al., 2019b) and AT-like domains (Santin and Moncalian, 2018; Hayashi et al., 2020a) have been confirmed by *in vitro* experiments. Among them, it has been confirmed that either two KS domains (Hayashi et al., 2019a; Santín et al., 2020) located in different subunits or two different types of DH domains (Hayashi et al., 2019b) have different selectivity for acyl-ACP substrates. However, in eukaryotic PUFA synthases, only the AT domain has been evaluated *in vitro* (Hayashi et al., 2020a; Almendariz-Palacios et al., 2021); hence, it is necessary to verify the respective catalytic activities and substrate recognition of the other domains. Specifically, there are two ER domains located in eukaryotic PUFA synthase, and their functions are likely to have some differences (Ling et al., 2020; Yang et al., 2021). *In vitro* assay may help identify their functional differences.

#### Structure analysis

The function of a protein is closely related to its structure, and the analysis of protein structure can help understand its catalytic mechanism more clearly. Therefore, the structural analysis of eukaryotic PUFA synthase will become increasingly important in future studies. In research on prokaryotic PUFA synthases, Trujillo et al. showed that the tandem ACP domains in *P. profundum* are relatively independent and form a beads-on-a-string configuration with high flexibility through small-angle X-ray scattering (SAXS) technology (Trujillo et al., 2013). Santí et al. Santín et al. (2020) reported the X-ray crystal structure of the KS-CLF didomain of *M. marina* and predicted ACP and acyl-chain binding sites of the KS-CLF didomain. Zhang et al. reported the 1.998 Å-resolution crystal structure of the ER domain in *S. piezoolerans* and analyzed the inhibition and catalytic mechanisms of the ER domain by docking NADP and a possible competitive inhibitor (Zhang et al., 2021). However, there is no report on the structures of domains in eukaryotic PUFA synthases. Furthermore, the architectures of iterative type I PKS and FAS have been reported (Herbst et al., 2018), indicating that it may be feasible to elucidate the entire structure of eukaryotic PUFA synthase in the future.

In addition to directly solving the structure of domains, computational methods can also be used to predict the structure of the protein. For example, homology-modeling servers such as SWISS-MODEL (Waterhouse et al., 2018) and Phyre2 (Kelley et al., 2015) can predict the structure of proteins by relying on structures of known proteins with high similarity, and more recently, AlphaFold2 (Jumper et al., 2021) has been used with a machine learning approach for the *de novo* structure prediction of proteins with atomic accuracy. Several reports mentioned the structures of eukaryotic PUFA synthase domains predicted by homology modeling methods, such as the three DH domains (Man et al., 2021) and the CLF domain (Li et al., 2018). The development of bioinformatics and computational biology studies has provided quick and convenient tools for revealing the catalytic activity and enzymatic modification of PUFA synthases.

### Modification of eukaryotic PUFA synthases

To increase the content of specific PUFA, the modification of eukaryotic PUFA synthases should be gradually carried out while elucidating the mechanisms of eukaryotic PUFA synthases. Some strategies, such as domain swapping and mutagenesis, can be used to modify eukaryotic PUFA synthases.

#### Domain swapping

Domain swapping is a common strategy to engineer the activities and functions of enzymes, and the products are likely to change by replacing domains with similar domains from other enzymes. In studies on prokaryotic PUFA synthases, the major PUFA product could be exchanged by exchanging KS-CLF didomain of DHA synthase with the corresponding domain of EPA synthase in *E. coli* (Hayashi et al., 2019a); the DH_FabA_ domains of EPA synthase were replaced with those of ARA synthase and produced ARA in *E. coli* (Hayashi et al., 2019b); Gemperlein et al. recombined the PUFA synthases of myxobacteria with different PUFA profiles, and heterologous expression of these chimeric PUFA synthases yielded different yeast strains with specific PUFA production profiles at promising yield (Gemperlein et al., 2019). Although the product profiles of eukaryotic PUFA synthases are almost the same, those of prokaryotic PUFA synthases are more diverse. Therefore, it is possible to change the catalytic activities and product profiles of eukaryotic PUFA synthases by replacing specific domains with corresponding prokaryotic domains. For example, Ren et al. (Ren et al., 2018) and Yang et al. (Yang et al., 2021) attempted respectively to replace the AT domain or ER_B_ domain with the corresponding domain from *Shewanella* in different *Schizochytrium* strains, and both increased the EPA content in PUFA product. But in fact, their genetic manipulation led to the destruction of the corresponding subunits of PUFA synthase, so their domain replacement works were not perfect. We believe that with the improvement of the gene manipulation strategies, there will be some better ways to achieve domain replacement.

#### Mutagenesis

With the increased level of structural elucidation of eukaryotic PUFA synthases, it gradually becomes possible to engineer PUFA synthase by random mutagenesis and site-directed mutagenesis. Utilizing random mutagenesis and screening, Hayashi et al. demonstrated that the amino acid mutation of F65L, F230L and I231T, located in the KS_B_ of *Aurantiochytrium* eukaryotic PUFA synthase, can change the main final product from the main DHA (EPA/DHA = 0) to the main EPA (EPA/DHA=1.07) in *E. coli* (Hayashi et al., 2019a). In addition, site-directed mutagenesis is also a precise way to introduce molecular diversity with less potential for global disruption of the protein architecture (Drufva et al., 2020). For instance, targeted point mutagenesis to residues in the PKS could alter domain specificity or selectivity, affect protein stability and interdomain communication, and promote more complex catalytic reactivity (Drufva et al., 2020). There are also examples in the study of PUFA synthase. For example, the mutation (E665A or R705K, located in the CLF domain) of *M. marina* DHA synthase completely lost the DHA production capacity and produced trace amounts of EPA in *E. coli* (Naka et al., 2019). Therefore, searching for possible mutation sites and carrying out targeted mutations are also the direction of eukaryotic PUFA synthase engineering in the future.

### Expanding the molecular biology toolbox in native hosts

At present, the genetic manipulation of eukaryotes that possess PUFA synthases is still challenging, which makes it difficult to modify and apply the eukaryotic PUFA synthase pathway. Expanding the molecular biology techniques of these eukaryotes may start from at least two aspects: developing efficient genetic transformation methods and establishing efficient genetic manipulation tools.

Until now, there has been no genetic transformation method that is universally applicable for PUFA-producing eukaryotes. In thraustochytrids, electroporation is the most common strategy for transformation (Wang et al., 2019; Han et al., 2020; Han et al., 2021; Rau and Ertesvag, 2021; Shi et al., 2021), and particle bombardment (Metz et al., 2009) and *Agrobacterium*-mediated transformation (Huang et al., 2021) have also been reported. To make the transformation more efficient, it is important to optimize the operating conditions according to different strains; in addition, other methods, such as bacterial conjugation (Sreenikethanam et al., 2022) and cell-penetrating peptide (CPP) transport (Rau and Ertesvag, 2021), should also be considered. For screening transformants, it has been reported that resistance genes for bleomycin, neomycin, hygromycin, cycloheximide and paromomycin (Wang et al., 2019; Du et al., 2021) can be used as selectable markers. However, the resistance to corresponding antibiotics varies greatly among different strains; thus, it is necessary to determine the inhibitory concentration specifically for different strains.

Additionally, other gene elements, such as promoters and terminators, for gene manipulation of PUFA-producing eukaryotes are limited. Rau et al. introduced some endogenous and exogenous promoters and terminators used in thraustochytrids (Rau and Ertesvag, 2021). To perform more complex genetic manipulations, more available gene elements need to be mined in the future. To achieve multigene expression under genetic element-restricted conditions, single multi-cistronic transcription units were constructed using 2A self-cleavage peptides in several thraustochytrid strains (Wang et al., 2020b; Rau and Ertesvag, 2021). Sun et al. reported the application of Cre/loxP site-specific recombination (Sun et al., 2015) to eliminate the antibiotic resistance gene in *A. limacinum* OUC88, which can reduce the number of different resistance genes needed in multistep genetic manipulation. In addition, most studies utilize homologous recombination for the gene integration of thraustochytrids, but the efficiency is low, and random integration still exists (Sakaguchi et al., 2012; Rau and Ertesvag, 2021). To achieve genetic manipulation at the nucleotide level, methods for site-directed integration and mutagenesis need to be developed. For example, the efficiency of specific gene knock-in by homologous recombination increased more than 10-fold by combining the clustered regularly interspaced short palindromic repeats (CRISPR/Cas9) system in *Aurantiochytrium* sp (Watanabe et al., 2021). Other gene-editing tools, such as zinc-finger nucleases (ZFNs), meganucleases (MNs), and transcription activator-like effector nucleases (TALEN)-mediated genome editing, have been used in many microalgae (Kumar et al., 2020), these tools can also be tested in PUFA-producing eukaryotes.

### Heterologous expression and metabolic engineering

Although the native hosts of eukaryotic PUFA synthase have high industrial value, their genetic engineering remains a challenge. Therefore, the heterologous expression of eukaryotic PUFA synthase in an appropriate host is also an option for PUFA production. At present, eukaryotic PUFA synthases have been expressed in *E. coli*, *Brassica napus* (Walsh et al., 2016) and *Arabidopsis*, and corresponding PUFAs have been detected in product (Table 1). In addition to these strains, some industrial microorganisms can also be used as chassis. For example, *Lactococcus lactis, Myxococcus xanthus, Pseudomonas putida* and *Yarrowia lipolytica* are used as chassis for the heterologous expression of prokaryotic PUFA synthase. (Table 3).

Heterologous expression of PUFA synthase and the production of PUFAs can be improved by using synthetic biology approaches such as sequence design, promoter selection and engineering, and operon design. Meesapyodsuk et al. expressed *Thraustochytrium* PUFA synthase in *E. coli* and found that the organization of three genes as one operon produced higher DHA and DPA than the expression of genes in three plasmids (Meesapyodsuk and Qiu, 2016)*.* Gemperlein et al. applied many strategies such as gene optimization and promoter engineering to better express myxobacterial PUFA synthases in *Pseudomonas putida* (Gemperlein et al., 2014; Gemperlein et al., 2016) and *Y. lipolytica* (Gemperlein et al., 2019), which improved the production of PUFAs and provided references for heterologous expression of eukaryotic PUFA synthase.

In addition, many metabolic engineering strategies can also be used to improve the PUFA production, for example, optimization of the precursor supply and reduction of PUFA catabolism. The key precursor of PUFA synthesis is malonyl-CoA, improving the synthesis of malonyl-CoA and weakening competitive pathways such as the FAS pathway can make more malonyl-CoA direct to the PUFA synthase pathway. β-oxidation of fatty acids will lead to a decrease in the production of major PUFA. Some researchers chose to knock out the *fadE* gene when expressing PUFA synthase from *Schizochytrium* in *E. coli*. (Hayashi et al., 2016). These metabolic engineering strategies can also be applied to the natural hosts of PUFA synthase (Chi et al., 2021).

## Conclusion

Some eukaryotic microalgae can accumulate a large amount of PUFAs and are gradually used for the industrial production of PUFAs. Compared with the desaturase/elongase pathway, the PUFA synthase pathway is simple and efficient, gradually attracting more attention from their potential application. However, the biosynthesis of PUFAs involves the cooperation of multiple domains of PUFA synthase, and the synthesis mechanism is complex and less clear. In this article, we first summarize the identification of PUFA synthases in eukaryotes and then review the functional studies of each domain and propose a possible process for the synthesis of PUFAs *via* the eukaryotic PUFA synthase pathway. In addition, we discuss the similarities and differences between the eukaryotic PUFA synthase pathway and the prokaryotic PUFA synthase pathway. Finally, we propose several new strategies that can be used to expand the research on the eukaryotic PUFA synthase pathway, such as identifying more eukaryotic PUFA synthases, deepening the research on the synthesis mechanism, modifying the eukaryotic PUFA synthase and expanding the molecular biology toolbox of PUFA-producing eukaryotes. This article provides a good reference for further investigation and engineering of the PUFA synthase pathway in eukaryotes and utilization of eukaryotic PUFA products for the maximal benefits of human beings.
